# Recent developments in the probiotics as live biotherapeutic products (LBPs) as modulators of gut brain axis related neurological conditions

**DOI:** 10.1186/s12967-022-03609-y

**Published:** 2022-10-08

**Authors:** Duygu Ağagündüz, Feray Gençer Bingöl, Elif Çelik, Özge Cemali, Çiler Özenir, Fatih Özoğul, Raffaele Capasso

**Affiliations:** 1 Department of Nutrition and Dietetics, Gazi University, Faculty of Health Sciences, 06490 Ankara, Emek Turkey; 2 Department of Nutrition and Dietetics, Burdur Mehmet Akif Ersoy University, İstiklal Yerleşkesi, 15030 Burdur, Turkey; 3 Department of Nutrition and Dietetics, Kırıkkale University, 71100 Kırıkkale, Merkez Turkey; 4 Department of Seafood Processing Technology, Faculty of Fisheries, Cukurova University, 01330 Balcali, Adana Turkey; 5 Department of Agricultural Sciences, University of Naples Federico II, 80055 Portici, NA Italy

**Keywords:** Probiotics, Live biotherapeutic products (LBPs), Gut brain axis, Neurodegenerative diseases, Safety

## Abstract

Probiotics have been defined as “living microorganisms that create health benefits in the host when taken in sufficient amounts. Recent developments in the understanding of the relationship between the microbiom and its host have shown evidence about the promising potential of probiotics to improve certain health problems. However, today, there are some confusions about traditional and new generation foods containing probiotics, naming and classifications of them in scientific studies and also their marketing. To clarify this confusion, the Food and Drug Administration (FDA) declared that it has made a new category definition called "live biotherapeutic products" (LBPs). Accordingly, the FDA has designated LBPs as “*a biological product that: i)contains live organisms, such as bacteria; ii)is applicable to the prevention, treatment, or cure of a disease/condition of human beings; and iii) is not a vaccine*”. The accumulated literature focused on LBPs to determine effective strains in health and disease, and often focused on obesity, diabetes, and certain diseases like inflammatory bowel disease (IBD).However, microbiome also play an important role in the pathogenesis of diseases that age day by day in the modern world via gut-brain axis. Herein, we discuss the novel roles of LBPs in some gut-brain axis related conditions in the light of recent studies. This article may be of interest to a broad readership including those interested in probiotics as LBPs, their health effects and safety, also gut-brain axis.

## Introduction

One of the most popular topics and research areas of recent years is the microbiome, microbiome modulation and factors that modulate the microbiome, especially nutrition. In this regard, the technological developments created by the 4th Industrial Bio-Revolution and especially the technological developments in the field of multiomics, such as The Human Genome Project (HGP), National Institutes of Health (NIH) Human Microbiome Project, European Metagenomics of the Human Intestinal Tract (MetaHIT) genome and microbiome projects play an important role [1–4] In these projects, microbiome characterization has been made and continues to be done with technologies such as 16S rRNA-encoding gene (16S) and metagenomic shotgun sequencing in different parts of the human body with multiomic technologies [1, 4].

The aggregate genomes of microorganisms in a specific habitat are referred to as the microbiome. The microbiome has been thought of as a virtual organ of the body for some time [5]. Berg et al. [6] defined the microbiome as “*It is a characteristic microbial community occupying a reasonable well-defined habitat which has distinct physiochemical properties*” [6]. In addition, unlike the microbiome, the microbiota was defined by the same researchers from a different perspective like “ *It consists of the assembly of microorganisms belonging to different, while “their theatre of activity” includes microbial structures, metabolites, genetic elements, and relic DNA embedded in the environmental conditions of the habitat*” [6].

Determining the structure and functional capability of the microbiome in health and disease, as well as the variables that influence it, is critical [7]. Microbiome-microbiota is a very dynamic ecosystem and can be affected by many modifiable and unchangeable factors for example genetic factors, age, geography, drug treatments [8]. Nutrition, which is an important source and modulator of biotics, is one of the most important modifiable factors that manipulate microbial diversity, composition and stability, affect the composition of microbiome and microbiota, and have the potential for therapeutic use depending on some factors [9]. Also antioxidants (vitamins, polyphenols etc.) are effective on gut microbiota. It has been stated that antioxidants reduce inflammation of gut microbiota, affect microbiota composition, intestinal mucosal barrier, short -chain fatty acids production and effect on the immune system [10, 11].

At this point, probiotics, metabolites of probiotics and modulators of probiotics play an important role. However, today, there are some confusions about traditional and new generation foods containing probiotics and some naming of some supplements, their use in scientific studies and their marketing [12]. In order to resolve this confusion, the Food and Drug Administration (FDA) declared that it has made a new category definition called "live biotherapeutic products" (LBPs) and the European Pharmacopoeia (Ph. Eur.) urgently determined the requirements for this new drug category [13, 14]. Accordingly, the FDA has designated LBPs as “*a biological product that: i) contains live organisms, such as bacteria; ii) is applicable to the prevention, treatment, or cure of a disease or condition of human beings; and iii) is not a vaccine*” [13]. European Pharmacopoeia (Ph. Eur.) defined LBPs as “medicinal products containing live micro-organisms such as bacteria or yeasts for human use” [14].

Although there is a literature focused on traditional probiotics, especially lactic acid bacteria (LAB), to determine effective strains in disease and health, such studies fall short of LBPs and often focus on obesity, diabetes, and certain diseases for instance Inflammatory bowel disease (IBD) [8]. However, the microbiome and microbiota play a significant role in the ethology of diseases that continue to be associated to stress in the modern world and age day by day [12]. Because there is a connection between the gastrointestinal system and the central nervous system (CNS). This link, which plays an important role in the pathogenesis of related diseases, is mediated by inflammatory cytokines, vagus nerve, neurotransmitters, and hypothalamic–pituitary–adrenal axis (HPA) [15]. There is growing evidence that LBPs affect the gut-brain axis, which in turn affects disorders related to the axis.

Gut-brain axis related conditions refer to many conditions from psychological disorders such as depression, anxiety, stress, bipolar disorder, and schizophrenia to neurological problems such as autism, Alzheimer's and Parkinson's. Current literature continues to present studies on the relationship between psychological conditions and LBPs [16–19]. It is accepted that the gut microbiota has a role in regulating psychological health in addition to physical health through the gut-brain axis. Besides negative changes in the intestinal microbiota may cause psychological disorders [20]. As with psychological disorders, the alteration of gut-brain axis interactions has been advocated as a potential cause of some neurological diseases [21]. In this review article, promising roles, mechanisms of action and possible safety issues of LBPs in gut-brain axis related neurological conditions are discussed in the light of current human and animal studies.

## Gut–brain axis related neurological conditions

### Autism

Autism is defined by social communication and interpersonal difficulties as well as limited repetitive behavior, activities, and interests. Defination of autism, according to the 5th Edition of the Diagnostic and Statistical Manual of Mental Disorders (DSM-5) as a condition that occurs with abnormalities in social communication and interaction and with repetitive, limited behavioral patterns or activities [22]. According to WHO-2018, approximately one in every 160 kids worldwide has autism. In the United States of America (USA), autism affects about one out of every 54 children nowadays [23]. Boys are four times as likely than girls to have autism [24]. The reason for this situation is not clear, but it is thought to be among the possibilities that it is related to estrogen and testosterone levels. Disruption of the transsulfuration pathway of testosterone [25], neuroprotective property of estrogen, and modulation of the gut microbiome [26] are suggested as reasons for the prevalence of autism in men.

Currently, there is no definitive treatment routinely used for autism. Physical therapy, cognitive education and sensory integration are among the treatments applied. According to new research, altering the gut microbiome may be a successful treatment for childeren with autism. Probiotics, prebiotics, microbiota transfer therapy, fecal microbiota transplantation (FMT), and various dietary treatment methods are gaining importance [27].

Causes of autism include genetic and environmental factors (for example oxidative stress, parental age, fetal infections, and fetal testosterone levels). Nutritional deficiencies due to selective eating behaviors of individuals with autism also have a significant influence in autism [28, 29]. Gastrointestinal symptoms including constipation, diarrhea, reflux, vomiting, discomfort, abdominal pain, gas, and unusually foul-smelling stools are common in autism [30]. It is stated that one of the main factors of gastrointestinal dysfunction in autism is "leaky gut syndrome" [27]. The epithelial cells are oblong with little paracellular space between them. Antigenic materials taken into the body cause inhibition of some enzymes, causing epithelial cells to become "round", which creates a significant increase in paracellular space. This situation, in which intestinal permeability is increased, may cause food-derived peptides to enter the circulation, as well as bacterial metabolites. These antigenic materials may trigger immune responses that influence neuronal signalling or cause the material to interact directly with the peripheral nervous system when they enter the circulation. In the pathophysiology of autism, increased intestinal permeability is assumed to be the link between the gut and the brain. [31]. In addition, it is stated that bacterial toxins and metabolites may cause increased oxidative stress and deterioration in detoxification mechanisms in individuals. [32].

Dysbiosis in the microbiota can be caused directly by certain genetic and environmental risk factors. Dysbiosis is increasingly being recognized as a feature of autism. [23]. The two dominant bacterial strain in the healthy human microbiota are the phyla *Bacteriodetes* and *Firmicutes* [33]. It was observed that *Bacteroidetes* and *Proteobacteria* were higher and *Actinobacteria* and *Firmicutes* were lower in a group of children with autism [34]. Table 1 lists the alterations in the microbiome of people with autism.

**Table 1:** Change in microbiota composition in autism

Increments	Descendants	Reference
*Bacteroidetes* *Bacteroides vulgatus* Desulfovibrio spp.	–	[33]
–	*Prevotella* *Coprococcus Veillonellaceae*	[34]
*Lactobacillus*	*Bifidobacter*	[35]
–	Sutterella spp.	[36]
*Clostridium histoliticum*	*Clostridium, cluster I* an*d II↓,*	[37]
Desulfovibrio spp. Lactobacillus spp	*Bacteroides/Firmicutes* ratio	[38]
*Clostridium, boltae,* *Clostridium, cluster I and XI*	–	[39]

In the presence of autism, there is a generally less diverse microbiota. Dysbiosis is stated to be present when autism is diagnosed, but a defined microbial signature for autism has not been identified. Reasons for uncertainty include methodological changes in symptom severity, lifestyle, comorbid conditions, medical history, and inherent heterogeneity of autism cohorts [34].

Low-grade systemic inflammation, increased intestinal permeability, and neuroinflammation are all symptoms of dysbiosis. Between the ENS, CNS, ANS, and HPA axis, there are complex, bidirectional integrated signalling networks that make up the gut-brain axis [40, 41]. The prefrontal cortex, hypothalamus, and limbic system among other emotional and cognitive brain regions, have been demonstrated to link with gastrointestinal function [42]. These processes can be influenced indirectly by the gut microbiome's metabolites and inflammatory mediators and directly by vagal stimulation. It has been discovered that the renin-angiotensin system has a role in the pathogenesis of associated illnesses as well as the modulation of brain function. High levels of oxidative stress, apoptotic pathways, and neuroinflammatory diseases are brought on by excessive activation of the ACE/Angiotensin II/Angiotensin type-1 receptor (AT-1) axis [43]. Meanwhile, it has been demonstrated that the pathophysiology of ASD is functionally related to immune system malfunction and an excess of reactive oxygen species (ROS) [44].Therefore, possible risk factors for ASD can be suggested for ACE gene polymorphisms. Studies are required to associate this mechanism with LBP, no studies were found.

SCFAs and tryptophan, the precursor to serotonin, are two bioactive metabolites produced indirectly by the gut microbiome as byproducts of cellular metabolism [45]. Bacterial metabolites associated with autism include serotonin and SCFAs. Tryptophan, one of the essential amino acids, is the precursor of the neuroinhibitor serotonin. Almost 90.0% of serotonin is produced by tryptophan, which is synthesized by the gut microbiota [46]. Given this contribution of the gut microbiota to serotonin production, overgrowth of certain types of bacteria can lead to an overproduction of serotonin in the gut and excessive consumption of tryptophan. This could potentially increase local gut serotonergic effects, leading to the mood and cognitive impairments seen in autism [46, 47]. Serotonin is produced by some strain of *Lactobacillus, Streptococcus* and *Lactococcus*. Increased serotonin synthesis caused by the microbiota could deplete tryptophan, contributing to the hyperserotonemia seen in autism [48]. The microbial strain that occurs more more commonly in children with autism are propionate producers for instance *Bacteroidetes, Clostridia,* and *Desulfovibrio* strain [33, 35, 38]. SCFAs can have neurotoxic effects when they reach the brain, and it is stated that propionate, in particular, can cause autism-like behavior in animal models [49]. The microbiome produces butyrate, which helps to maintain the integrity of the intestinal epithelium and create T-regulatory (Treg) cells [50]. By limiting the body’s ability to regulate the immune system, changes in SCFA levels can increase proinflammatory chemicals entering the systemic circulation and exacerbate neuroinflammation. By affecting the permeability of the intestinal epithelial barrier and the blood–brain barrier, a dysbiotic gut microbiome can cause systemic and CNS inflammation [51, 52].

**Fig. 1:**
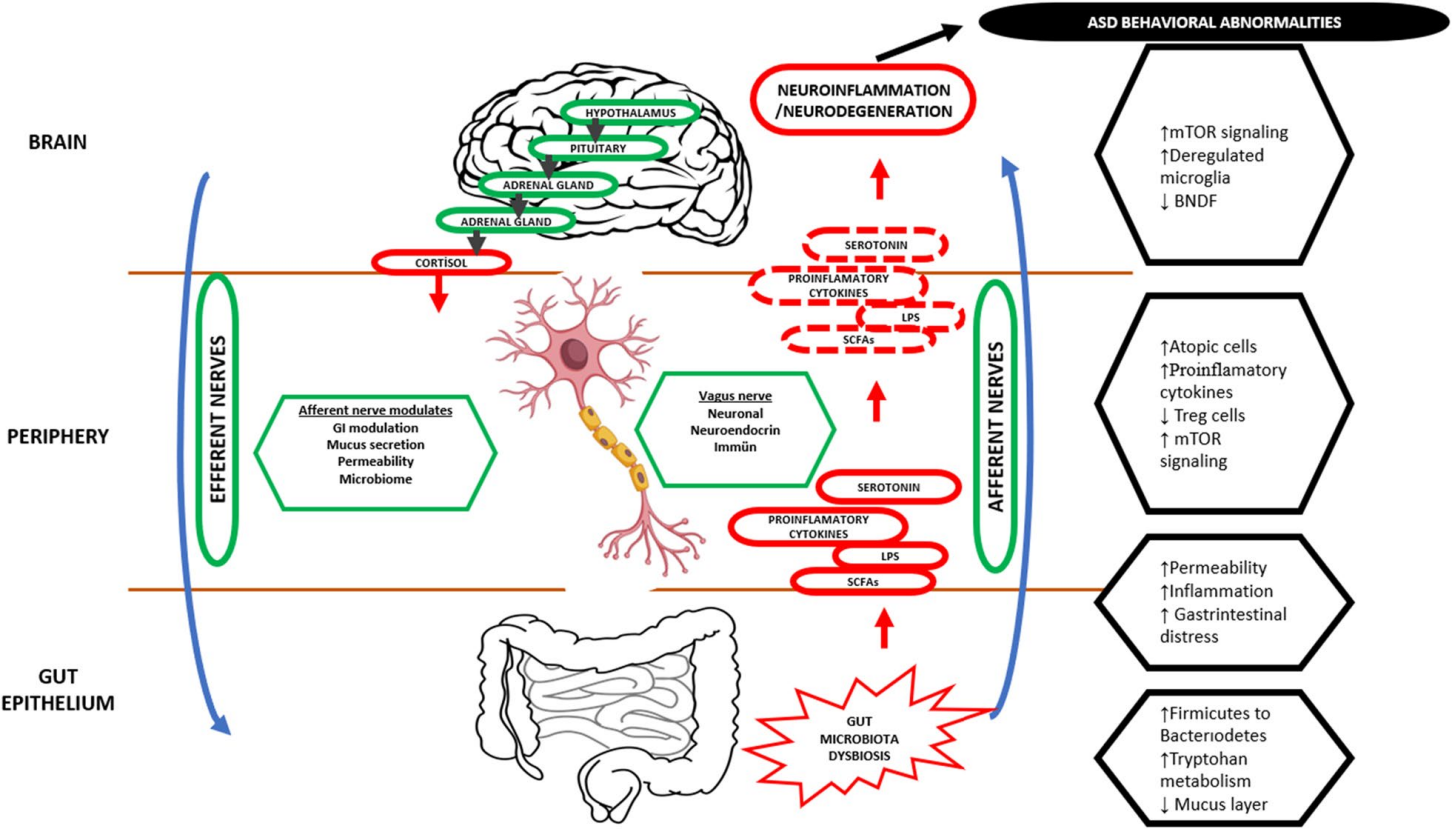
Relationship between microbiota, gut-brain axis and autism (adapted from references [23] and [56])

Afferent connections directly connect the gut wall to certain brainstem nuclei that can be activated by changes in microbiome composition, and efferent connections connect sympathetic glutamatergic neurons in the CNS to the gut are found in the vagus nerve. This, in turn, may regulate gastrointestinal function and thus microbiome composition [53]. The gut microbiome can have tangible, far-reaching effects on neurological function because of these connections. Autism is linked to these pathways. Dysfunction of the HPA, especially autonomic dysregulation, including cortisol dysregulation and reduced vagal tone, has been linked to autism [54]. ANS function has been associated with key features of autism deficits in social behavior, language skills, and cognitive delay [55]. Figure 1 presents several explanations for the association between the microbiome, gut-brain axis, and autism.

Eliminating the imbalance in the microbiota-brain axis is seen as a potential target in the treatment of autism [23, 57]. It is thought that LBPs may be effective on autism symptoms through the intestinal microbiota. To better understand the effects of LBPs, a recent study on animals with autism assessed changes in ASD-related behaviours, gut microbiota, and systemic and cellular metabolism. The study used the *Lacticaseibacillus rhamnosus* HA-114 and *Liglactobacillus salivarius* HA-118, which were formerly included under the Lactobacillus genus. Through social contact, the diversity of gut microbes, and the constructive manipulation of neuroactive signalling molecules along the microbiota-gut-brain axis, this study established the psychobiotic potential of *L. rhamnosus* HA-114. Although *L. salivarius* HA 118 had a beneficial impact on social behaviour, it had no impact on gut microbiota and neuroactive metabolites [58]. In a different animal study, it was shown that treating mice with *L. reuteri* reduced repetitive behaviour in both male and female Shank3 KO mice and attenuated antisocial behaviour, especially in the males. Additionally, it has been demonstrated that L. reuteri therapy alters the protein and gene expression levels of GABA receptors in various parts of the brain [59]. This result is consistent with previous study showing a relationship between Lactobacillus, autism-related behaviors and GABAergic function [60].

Some recent studies on this subject in humans are given in detail in Table 2. The bacteria strain, *Lactobacillus Acidophilus, Lacticaseibacillus rhamnosus, Lactiplantibacillus Plantarum, Lacticaseibacillus casei, Lactobacillus delbrueckii subsp. bulgaricus, Bifidobacteria longum, Bifidobacterium infantis* and *Bifidobacterium breve* used in the studies are very diverse [61–64]. Studies using a capsule containing 8 strains of live bacteria [49, 51] and using Lactobacillus Acidophilus, *Lacticaseibacillus rhamnosus* and *Bifidobacteria longum* [50] generally showed improvement in gastrointestinal symptoms compared to the control group. Autism symptoms were assessed using a variety of scales in the research, and autism symptoms definitely improved, but because several probiotics were given, it is unclear which probiotic had the greatest effect [61, 63, 64]. The fact that Santocchi was evaluated in proinflammatory cytokines, unlike the others, although no significant changes are observed [51], it is important for understanding the metabolism, more studies are needed on this subject. The high proportion of males in the samples of the given studies is consistent with the higher incidence of the disease in males [48–51]. Most of the studies on autism in Table 2 are randomized controlled studies that yield the most scientifically valuable results according to the scientific evidence pyramid [48, 49, 51]. The fact that the duration of use of LBPs was different in the studies examined, except for one study [49]. Except for two studies (which are microbiota changes [49, 50]), it was not specified how long the observed positive outcome persisted. The food consumption of individuals, which is an important factor in the composition of the microbiota, was not included in the studies examined. It can be a guide for future studies. There is no standardization between studies on gastrointestinal symptoms and autism severity assessment scales, which causes the effects to not be interpreted clearly. One of the most important shortcomings in understanding metabolism is that the results are not associated with any metabolic parameter.

**Table 2:** Novel certain human clinical trials on live biotherapeutic products (LBPs) in gut brain axis related conditions

Gut brain axis related conditions	LBPs	Subjects	Dose	Intervention duration	Results	Reference
Autism	*Lactobacillus Acidophilus* *Lacticaseibacillus rhamnosus* *Bifidobacteria longum*	ASD cases (n:30) %63.3 male Controls (n:30, matched gender and age) 5–9 years old Prospective, open-label, case–control study	Probiotic mixture each gram contains 100 × 10^6^ CFU Obtainable as a powder to be diluted in water Once-daily 5 g/day	3 months	Pre-intervention *Bifidobacteria* levels less in ASD feces were lower than in the control group Bifidobacteria and Lactobacillus levels increased after probiotic intervention in in ASD children Autism Treatment Evaluation Checklist (ATEC) overall scores decreased. It demonstrates that the severity of ASD symptoms decreased Overall GI symptom severity was reduced (constipation, gas, stool consistency, and abdominal pain)	[63]
Autism	*Lactiplantibacillus Plantarum PS128*	Aged 7–15 years n: 71, (%100 male) (E: 36, C: 35) Randomized, placebo-controlled, double-blind study	3 × 10^10^ CFU with microcrystalline cellulose Placebo: Microcrystalline cellulose 1 capsule Daily	4 weeks	Opposition/defience behaviors improved after treatment wirh *Lactiplantibacillus plantarum PS128* Observing that younger children (7–12 years old) benefit more than older children (13–15 years old), the intervention appears to have an age-related effect	[61]
Autism	Visbiome® (formerly VSL#3) Four strains of *Lactobacilli* *Lacticaseibacillus casei,* *Lactobacillus delbrueckii subsp. bulgaricus,* *Lactiplantibacillus plantarum,* *Lactobacillus acidophilus* Three strains of *Bifidobacteria Bifidobacterium infantis Bifidobacterium breve* *Bifidobacterium longum* One strain of *Streptococcus Thermophiles*	Aged 3–12 years N:13, (%60 male) (Group 1:6, Group 2: 4) Group 1: 8-week probiotic + 3-week washout + 8-week placebo Group 2: 8-week placebo + 3-week washout + 8-week probiotic Randomised, cross-over pilot, placebo-controlled study	90 × 10^10^ CFU/packet Dose: 1/2 packet Twice a day (weeks1–4) Option to increase to 1packet twice a day at (week 5–15) Placebo: matched but ingredients not stated	19 weeks (8 weeks, 3-week washout, 8 weeks)	GI symptoms that were aimed at the parents improved significantly There was no change in the gut microbiome diversity or strain family-level composition Probiotics have a clear transport effect The probiotic effects lasted throughtout the entire 3- washout period	[62]
Autism	Visbiome® (formerly VSL#3) Four strains of *Lactobacilli* *Lacticaseibacillus casei,* *Lactiplantibacillus plantarum,* *Lactobacillus delbrueckii subsp. bulgaricus, Lactobacillus acidophilus,* Three strains of *Bifidobacterium longum Bifidobacteria* *Bifidobacterium breve Bifidobacterium infantis* One strain of*Streptococcus Thermophiles*	18–72 months (4.2 years) (%84 male) n: 63 ASD treatment (n: 31) ASD Control (n: 32) Placebo controlled, randomised trial	450 billion CFU were present in each packet 2 packets/day in the first month of treatment and 1 packet/day in the following 5 months Placebo: 4.4 g of maltose + silicondioxide	6 months	There is no significant difference in the Total Autism Diagnostic Observation Schedule – Calibrated Severity Score (ADOS-CSS score) Plasma biomarkers (IL-6, TFN-α) and faecal calprotectin didn’t differ significantly Analysis of subgroups There was no GI group (n = 46) Total ASD severity scores ADOS and Social-Affect ADOS scores decreased GI group (n = 17) Between baseline and 6 months, the probiotic group exhibited a significant difference from the control group Reduced total GI severity, stool odor and flatulence Improved adaptive skills (Repetitive, Domestic, Coping skills) Scores from sensory profiles were normalized (improvements in 87 percent of probiotic group vs 28 percent of placebo group)	[64]
Alzheimer Disease	*Lactococcus lactis W19* *Lactobacillus acidophilus* *W22* *Lacticaseibacillus casei* *W56* *Bifidobacterium lactis W52* *Lacticaseibacillus paracasei* *W20* *Lactiplantibacillus plantarum W62, Bifidobacterium lactis* *W51* *Bifidobacterium bifidum W23 Ligilactobacillus salivarius W24*	Twenty (11 males, 9 females, aged 76.7 ± 9.6 years) Alzheimer’s disease patients	Omnibiotic Stress Repair (Allergosan, Graz, Austria)	28 days	The serum levels of kynurenine significantly increase as a result of probiotic supplementation BDNF levels did not change before or after taking probiotic supplements for 4 weeks After 4 weeks of probiotic administration, the RNA content of the fecal bacteria strain *Faecalibacterium prausnitzii* considerably increased, whereas the contents of *Clostridium cluster I* and *Akkermansia muciniphila* remained unaltered Zonulin concentrations dropped at the two time points just before and after taking probiotic supplements for four weeks	[65]
Alzheimer Disease	*Limosilactobacillus fermentum* *Lactiplantibacillus plantarum Bifidobacterium lactis* or *Lactobacillus acidophilus* *Bifidobacterium longum Bifidobacterium bifidum*	Forty-eight alzheimer disease individuals, with no comorbidities and aged 65–90 years Control (n: 23) Probiotic (n: 25)	Total dosage of 3 × 10^9^ CFU (2 capsules once a day)	12 weeks	There have been no significant changed Test Your Memory, total antioxidant capacity (TAC), glutathione (GSH), malondialdehyde (MDA), IL-6, TNF-α, IL-10, nitric oxide (NO), 8-hydroxy-2' -deoxyguanosine (8-OHdG) levels	[66]
Alzheimer Disease	*Bifidobacterium breve A1 (MCC1274)*	50–79 years 80 healty individuals with mild cognitive impairment (MCI) Probiotic group (n: 39) Placebo group (n: 40) A Randomized, Double-Blind, Placebo-Controlled Trial	2 × 10^10^ CFU	16 weeks	When compared with placebo in the probiotic group; Immediate memory, visuospatial/constructional score, delayed memory and Repeatable Battery for the Assessment of Neuropsychological Status (RBANS) were significantly improved	[67]
Alzheimer Disease	*Lacticaseibacillus rhamnosus GG*	52–75 year old adults were enrolled Probiotic group (n: 77) Placebo group (n: 68)	Culturelle Vegetarian Capsules containing a 10 billion CFU blend two capsules daily	90 days	In middle-aged and older persons with cognitive impairment, supplementation was associated with enhanced cognitive function	[68]
Parkinson Disease	*Lactobacillus acidophilus Bifidobacterium bifidum, Limosilactobacillus reuteri* *Limosilactobacillus fermentum*	Aged 50–90 yaers, had a Parkinson Disease diagnosis Probiotic (n: 30) Placebo (n: 30)	Each bacteria 2 × 10^9^ CFU/g	12 weeks	In probiotic group; The Movement Disorders Society-Unified Parkinson's Disease Rating Scale (MDS-UPDRS) was reduced Serum hs-CRP, MDA, insülin, HOMA-IR, triglyceride and very low-density lipoprotein-cholesterol (VLDL-C) levels were reduced and GSH levels were increased	[69]
Parkinson Disease	*Lactobacillus Acidophilus* *Limosilactobacillus reuteri* *Lactobacillus gasseri Lacticaseibacillus rhamnosus,* *Bifidobacterium bifidum, Bifidobacterium longum,* *Enterococcus faecalis, Enterococcus* *faecium*	Aged 40 years or older, had a Parkinson Disease diagnosis individuals Probiotic (n: 34) Placebo (n: 38)	Each probiotic capsule contained 10 billion CFU	4 weeks	The average number of spontaneous bowel movements (SBM) per week was increased The groups didn’t differ significantly in ters of fecal calprotectin changes from baseline to the end of the treatment Changes in stool consistency, constipation severity score, and quality of life related to constipation all showed significant improvements in the intervention group	[70]
Parkinson Disease	*Lactiplantibacillus plantarum PS128 (PS128)*	Aged 40–80 years had a Parkinson Disease diagnosis 8 female/17 male	Two capsules Daily (30 billion CFU per capsule)	12 weeks	Administration of PS128 was any significant impact on rigidity, tremor, PIGD subscores, or Mhys Significantly decreased Unified Parkinson’s Disease Rating Scale (UPDRS) motor scores and akinesia subscores PS128 use significantly decreased plasma myeloperoxidase levels and the urinary creatinine levels When comparing the scores of individuals with Parkinson’s Disease Questionnaire (PDQ-39), the single index score, mobility, activities of daily living, stigma and cognition significantly decreased after the 12-week supplementation	[71]

Firstly in animals, then in humans, to fully comprehend the LBPs mechanism of action in ASD, more study is required on the effects of specific probiotic treatment on immunological responses, brain activity and metabolism.

Considering that autism arises due to the adverse interactions of the gut, brain, and immune system, evaluation of neurotransmitters, bioactive bacterial metabolites and inflammatory markers as well as gastrointestinal symptoms and autism severity in future randomized controlled clinical trials with live biotherapeutic products, will contribute to both understanding the metabolism in humans with clearer evidence and understanding the effectiveness of the supplement used. Obtaining clearer and more consistent results from studies will make it clear whether the use of LBPs is effective. This will pave the way for the disease-specific use of these new generation products.

### Alzheimer's disease

The most prevalent form of dementia, Alzheimer’s disease is a progressive neurodegenerative condition marked by the accumulation of amyloid peptides (Aβ) in the brain [87]. According to the data of the Alzheimer's Association, it was stated that 6.2 million Americans were living with Alzheimer's dementia in the USA in 2021 and this number was estimated to be 12.7 million in 2050 [88]. The World Alzheimer's Disease 2021 report estimates that 55 million individuals worldwide already suffer from dementia, and that figure will rise to 78 million by the year 2030 [89].

Alzheimer's disease is characterized by the buildup of amyloid in the brain. Aβ peptides are synthesized from β-amyloid precursor proteins (APP). With the help of the β-secretase (BACE1) and γ-secretase complex, APP switches to the amyloidogenic route, whereas α-secretase is involved in the creation of the non-amyloidogenic pathway.

While Aβ accumulates in the extracellular, neutrophil clumps are formed in the intracellular [87, 90, 91]. Alzheimer's disease risk factors can be listed as age and gender, head injuries, cardiovascular diseases, lifestyle, environmental factors, diet, infection, genetic factors, obesity and other diseases such as diabetes [92]. Oxidative stress has an impact on how Alzheimer's disease develops. In case of increased oxidative stress, mitorchondial dysfunction, Aβ aggregation, disruption of membranes, molecular oxidation, hyperphosphorylation of tau protein (microtubule-associated protein) may occur. In addition, an increase in reactive oxygen strain is associated with an increase in the oxidation of DNA, proteins, and lipids [93, 94]. In addition, the disease was associated with an increase in inflammation and inflammatory cytokines. Increase in inflammatory cytokines may cause increased Aβ aggregation and tau phosphorylation and this can cause neurotoxicity and neurodegeneration as a result of neuroinflammation [95–97]. The renin-angiotensin system is one of the risk factors for Alzheimer's. In the cortical and hippocampal regions of Alzheimer's disease versus non- Alzheimer's disease brains, AT1 receptor expression increased in the hippocampus, whereas AT2 receptor expression remained essentially unaltered. In Alzheimer's disease brains, there were small decreases in ACE-1 protein levels in the cortex and hippocampus, along with slight increases in ACE-2 levels in the cortex [98]. In a meta analysis study containing 15 studies use of angiotensin II receptor blockers (ARBS) significantly decreased the risk of Alzheimer's disease and other forms of dementia [99]. AT1 receptor activation is linked to an increase in oxidative stress, anxiety and stress. Angiotensin (1–7), which consists of angiotensin 1 and 2, has been connected to the MAS (AT7) receptor and shows effects such as antioxidant, antiinflammatory, neurogenesis. At the same time, The muscularis mucosa, propria, small intestinal brush border, microvascular endothelium, and vascular smooth muscle cells are all areas of the gut that include Angiotensin (1–7) axis components [100, 101]. In a study Angiotensin (1–7) values and white matter hypointensities volumes were positively and significantly correlated in Alzheimer's disease patients, and Ang-(1–7) levels in plasma were significantly lower in Alzheimer's disease patients than in controls [102].

In addition to all these factors, dysbiosis in the microbiota is also associated with Alzheimer's Disease. Intestinal bacterial dysbiosis has been shown to be associated with altered intestinal permeability, systemic activation of the immune system, production and accumulation of bacterial Aβ fibrils in the brain, and increased neuroinflammation that contribute to Alzheimer's disease [103]. In a related study, it was found that the microbial diversity decreased, and its composition changed in people with Alzheimer’s Disease. It’s been established that *Bifidobacterium* and *Firmicutes* levels decrease and *Bacteroidetes* increase in the microbiome of individuals with Alzheimer's disease [104]. *Bacteroides* (enterotype I) strain have also been shown to be decreased in individuals with dementia [105]. In another study, it was shown that *Lactobacillus, Dorea, Bifidobacterium, Streptococcus, Blautia* and *Escherichia* strain increased and *Alistipes, Parabacteroides, Bacteroides, Sutterella* and *Paraprevotella* decreased in feces in Alzheimer's patients. *Escherichia* and *Lactobacillus* strain were increased and *Bacteroides* decreased in general in people with Alzheimer's and moderate cognitive impairment [106]. In addition, increased levels of LPS and proinflammatory cytokines associated with dysbiosis are associated with amyloid deposition [107]. It's been demonstrated that fecal SCFAs decrease, blood brain barrier function is impaired, and proinflammatory cytokines such as IL-1β, IL-6 and TNF-α increase in cognitive impairment [108].

A variety of factors, including dysbiosis, altered gut-brain axis, and changes in the microbiota, are linked to Alzheimer's disease. The state of dysbiosis generally causes an increase in intestinal permeability, Toll-like receptor (TLR) cell activation, bacterial amyloid formation, and the emergence of bacterial metabolites. This may result in weakening of the gut-brain barrier, production of inflammatory markers, and Aβ formation by gut bacteria [109]. The gut microbiota is a significant source of amyloid. In particular, amyloid is produced by *Escherichia coli* and helps bacterial cells bind together by forming a biofilm and resist destruction by physical or immune factors. Although bacterial amyloids differ from central nervous system amyloids in their primary structure, they share similarities in their tertiary structure. The change in its amount can also affect Aβ in the brain as a result of the change in the immune response, trigger the inflammatory response and increase the aggregation of other misfolded proteins such as α-synuclein [110, 111]. Gender is one of the factors affecting microbiota in Alzheimer's disease. In a study conducted in female and male wildtype (WT) and Tg mice, differences in behavioral and cognitive performances and short-chain fatty acids were observed between genders. When compared to WT-M, Tg-F, and Tg-M mice, butyrate concentration was higher in WT-F mice. Butyrate levels showed a positive correlation with working memory and object recognition, with WT-F mice having the highest values and Tg-F mice having the lowest. Differences in fecal microbiota composition were also found between different species and gender [112]. In a study in which *App*^NL−G−F^ male and female mice were supplemented with *VSL#3* probiotics. In *App*^NL−G−F^ female mice, probiotic feeding decreased Aβ plaque load and enhanced memory [113].

Both bacteria and their by-products (amyloid and LPS) can enter the brain and cause neuroinflammation. Additionally, the blood–brain barrier is severely compromised by bacterial translocation and the release of proinflammatory cytokines, which can also set off neuroinflammatory cascades. In the case of Alzheimer's disease, LPS are powerful activators of the TLRs and the AGEs receptor, which sustain chronic inflammation. Systemic inflammation induced by LPSs can affect the formation of Aβ, increase the permeability of the blood brain barrier, and decrease the synthesis and secretion of neurotrophic factors such as N-methyl D-Aspartate (NMDA) and BDNF receptors. Decreased BDNF levels and NMDA signal; It is associated with cognitive decline in addition to mood disorders. Neuroplasticity, which has been shown to be a key marker of disease, is influenced by both BDNF and NMDA [110, 114–117]. Proinflammatory cytokines are known to promote APP expression, upregulate β-secretase messenger RNA (mRNA), and increase Aβ formation in the hippocampus [118]. Studies in rats in Alzheimer's disease have shown that LPS increases TLR4, exacerbates cognitive impairment, and exacerbates neuronal apoptosis [119]. IL-1 overexpression led to an increase in tau phosphorylation. [120].

Alterations in the tryptophan-kynurenine metabolism have been linked to yet another connection between the microbiota and Alzheimer's disease. In this pathway, there are 4 main metabolites: quinolinic acid (QA), 3-hydroxykynurenine (3-HK), kynurenic acid and picolinic acid. In case of changes in the ratios of these metabolites, 3-HK and QA metabolites may become neurotoxic and cause microglia activation and cell death. Particularly, A group of essential tryptophan-metabolizing enzymes in the kynurenine pathway called indoleamine 2,3-dioxygenase 1 (IDO-1), is stimulated by the proinflammatory cytokines IFN-γ and TNF-α and has been found to settle in similar sites with Aβ plaques. The kynurenic acid and picolinic acid obtained in this pathway have neuroprotective effects [115, 121].

**Fig. 2:**
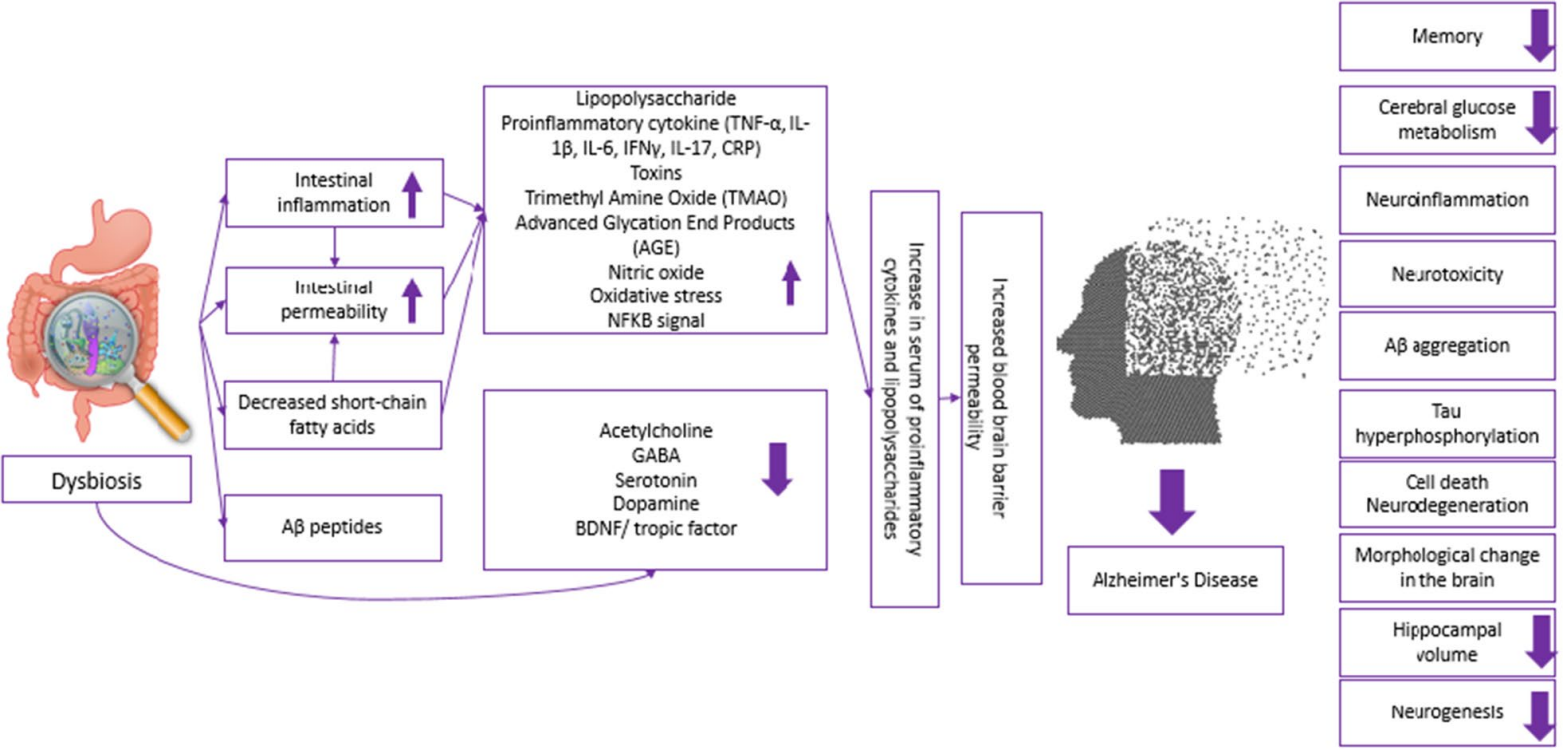
Possible effects of dysbiosis in the microbiota in Alzheimer's disease

The possibility that bile acids play a role in Alzheimer's disease is another mechanism demonstrating the connection between the microbiota and the disease. Bile acids are converted to secondary bile acids in the intestines. It has been suggested that the reduction in cognitive function may be partly due to the cytotoxic properties of deoxycholic acid, which can disrupt the blood brain barrier and penetrate brain tissue [115]. In addition, the synthesis of neurotransmitters such as dopamine, noradrenaline, acetylcholine, serotonin, GABA, and histamine take place by the microbiota. In the case of dysbiosis, a decrease in acetylcholine, GABA, serotonin levels, BDNF, neurogenesis and neuronal growth may occur. Increased nitric oxide levels increase oxidative stress [111, 122]. Figure 2 summarizes the relationship between dysbiosis in the microbiota and Alzheimer's disease.

It has been emphasized that probiotic/prebiotic supplementation, depending on the changes in the microbiota, may have a positive effect by reducing inflammation through the rearrangement of the microbiota, improving cognitive function, and reducing Aβ and tau proteins [123]. Human and animal studies on probiotic supplementation in Alzheimer's disease are given in Table 2 and Table 3. *Lactobacillus* and *Bifidobacterium* bacteria were generally used in both study types. Probiotics showed effects on kynurenine pathway, BDNF level, microbiota composition, inflammation, oxidative stress, cognitive performance, Aβ plaques, SCFAs levels [65–67, 72–75, 77, 78, 124]. Although the effect of probiotic supplementation in Alzheimer's disease on renin angiotensin system is not fully evaluated, Angiotensin (1–7)-expressing probiotic bacteria Lactobacillus paracasei (LP) raised serum serotonin and the neuroprotective biomarker 2-picolinic acid, kynurenine aminotransferase II mRNA expression, angiotensin (1–7) and reduced neuro-inflammatory gene expression in the pre-frontal cortex and serum angiotensin II levels [125, 126]. In another study conducted in obese mice, orally *Bifidobacterium longum* supplementation increased in Mas receptor expression and angiotensin converting enzyme 2 (ACE2) levels [127].

**Table 3:** Novel certain animal model studies on live biotherapeutic products (LBPs) in gut brain axis related conditions

Gut brain axis related conditions	LBPs	Subjects	Dose	Intervention duration	Results	Reference
Alzheimer Disease	*Lactiplantibacillus plantarum*	Eight-week-old C57BL/6 J mice (male, n = 60) transgenic (APP/PS1) Five group 1.WT = wild type 2.APP/PS1 mice 3.Memantine group 4. *Lactiplantibacillus plantarum* group 5.Memantine + *Lactiplantibacillus plantarum* group	1 × 10^9^ CFU/mL	12 weeks	Between APP/PS1 mice group and *Lactiplantibacillus plantarum* group; Neuroinflammation in the hippocampus was reduced (hippocampus IL-2, IL-17, TNF-α) Trimethylamine (TMA) and trimethylamine N-oxide (TMAO) levels were reduced and hepatic flavin monooxygenase (FMO) activity was increased while FMO3 levels remained constant in the liver The number of Αβ plaques in the hippocampus were decreased	[72]
Alzheimer Disease	*Lactobacillus acidophilus (1688FL431-16LA02), Limosilactobacillus fermentum (ME3), Bifidobacterium lactis (1195SL609-16BS01), Bifidobacterium longum (1152SL593-16BL03)*	Sixty male Wistar rats (weight 180–220 g, 8 weeks of age) 1. Control 2. CP: probiotics; S: sham; 3. Aβ: Alzheimer; 4.AP: Alzheimer-probiotics	2 g (1 × 10^10^ CFU/g)	8 weeks	Improved spatial memory Spending more time in the in the target quadrant No significant difference found superoxide dismutase (SOD), catalase (CAT) levels with probiotic supplementation The AP group had significantly much lower levels of malondialdehyde (MDA) than the the Aβ group Between Aβ and AP groups, total Lactobacillus and Bifidobacterium count increased The AP group’s escapelatency and travelled distance were significantly decreased in comparison to the the Aβ group	[73]
Alzheimer Disease	VSL#3 *(Lactiplantibacillus plantarum, Lactobacillus paracasei,* Lactobacillus delbrueckii sub sp. *Bulgaricus,* *Lactobacillus acidophilus, Bifidobacterium* *longum, Bifidobacterium breve,* *Bifidobacterium infantis, and Streptococcus salivarius substrain, thermophilus)*	C57BL/6 wild-type (WT) mice were compared to App^NL−G−F^ mice 1.WT 2.WT + VSL3 3.App^NL−G−F^ mice 4. A4.pp^NL−G−F^ mice + VSL3	0.32 × 10^9^ CFU bacteria/25 g mice	8 weeks	Following probiotic treatment App^NL−G−F^ mice displayed a significant increase in *Clostridia, Lachnospiracea* and *Akkermansia* genera The serum levels acetate, butyrate, lactate, isobutyrate and propionate were increased after probiotic supplementation Acetate and lactate concentrations in the hippocampus region were found to be elevated c-Fos immunoreactivity was increased after probiotic supplementation After probiotic treatment, Aβ, GFAP, and Iba-1 immunoreactivity didn’t affected The levels of Aβ in the hippocampus were unaffected by probiotic treatment Anxiety-like behavior was altered after Probiotic supplementation	[74]
Alzheimer Disease	*Lactiplantibacillus plantarum Bifidobacterium bifidum*	Forty male Wistar rats, weight of 280 ± 20 g 1.Control (healthy rats), 2. Receiving Aβ AD models (Aβ), 3. AD rats with MIIT (Aβ + MIIT), 4.AD rats fed *Lactiplantibacillus plantarum* and *Bifidobacterium bifidum* (Aβ + PROB), 5. Receiving both treatments for AD rats (Aβ + MIIT + PROB)	1 × 10^9^ CFU of each strain	8 weeks	No significant difference BDNF and choline acetyltransferase (CHaT) levels between Aβ and Aβ + PROB groups	[75]
Alzheimer Disease	*Lactobacillus acidophilus,* *Bifidobacterium bifidum Bifidobacterium longum*	Male Sprague–Dawley rats weighing 220∼250 g 1.Sham 2.Alzheimer 3.Alzheimer + Probiotic	500 mg probiotics [15 × 10^9^ colony-forming units (CFU)	6 weeks	Decreased escape latency significantly No significant difference in nitric oxide concentration The total cholesterol, triglyceride, and very low-density lipoprotein-cholesterol (VLDL-C) concentrations in the serum and paired-pulse facilitation (PPF) ratio were reduced Increase in field excitatory postsynaptic potentials (fEPSP)	[76]
Alzheimer Disease	*Bifidobacterium. bifidum BGN4* *Bifidobacterium. longum BORI*	C57BI/6 and 5xFAD mice 1.Control-BGN4/BORI group (n = 10), 2.Control + BGN4/BORI group (n = 10) 3.5xFAD-BGN4/BORI group (n = 10), and 4.5xFAD + BGN4/BORI group (n = 10)	1 × 10^9^ CFU in 0.2 ml sterile water	30 days	BDNF protein expression in the hippocampus was increased Amyloid-β42 positive cells were reduced in the hippocampus In cleaved caspase-3 positive cells were decreased Reduced neuronal death in CA3 and CA1 areas of the hippocampus The number of Map2 + /BDNF + neurons in the hippocampus were significantly increased AD-associated memory deficits were improved The expression of IL-17 and IL-6 was reduced	[77]
Alzheimer Disease	*Akkermansia muciniphila GP01*	APPswe/PS1dE9 (APP/PS1) double-transgenic mice WT mice were divided into two groups (n: 6 per group) while APP/PS1 mice were randomly divided into four groups (n: 10 per group)	5 × 10^9^ CFU of *Akkermansia muciniphila* in 200 µL sterile PBS	6 months	Aβ plaque deposits and Aβ levels were reduced in brains Impaired cognition and anxiety-related behaviors were improved Glucose homeostasis was regulated, and damage to the intestinal barrier was reduced Decrease in serum cholesterol and triglyceride levels Uncoupling protein 1 (UCP1) level was increased in brown adipose tissue	[78]
Alzheimer Disease	*Bifidobacterium* breve (*Bifidobacterium* breve NMG, *Bifidobacterium breve* MY, *Bifidobacterium* breve CCFM1025, *B*i*fidobacterium* breve XY, and *Bifidobacterium* breve WX)	Eighty 8-week-old, C57BL/6 J male mice Sixty-four mice 1.Control 2.Model-Aβ_1-42_ 3.Donepezil- Aβ_1-42_ 4.Aβ_1-42_ *Bifidobacterium* breve NMG 5.Aβ_1-42_ *Bifidobacterium* breve MY, 6.Aβ_1-42_ *Bifidobacterium* breve CCFM1025, 7.Aβ_1-42_ *Bif*i*dobacterium* breve XY 8.Aβ_1-42_ *Bifidobacterium* breve WX	10^9^ CFU/ml for oral administration	6 weeks	The treatment of *Bifidobacterium* breve NMG and CCFM1025 resulted in significant improvements in alternation behaviour as well as an increases in total arm entries *Bifidobacterium* breve treatment improves Aβ_1-42_-induced memory defects, CCFM1025, XY, and WX significantly reduced Aβ_1-42_-induced hippocampal accumulation in Aβ_1-42_ treated mice CCFM1025 treatment significantly improved synaptic plasticity and led to increased concentrations of BDNF, fibronectin type III domain containing 5 (FNDC5), and postsynaptic density protein 95 (PSD-95). Interestingly, all bifidobacteria strains raised BDNF concentrations of except MY Butyrate and acetate concentrations were found to be significantly decreased in AD mice, while propionate concentrations were significantly increased The concentration of acetate was significantly increased by 1025 and WX Butyrate concentrations in the feces of the CCFM1025-treated group were significantly increased	[79]
Parkinson Disease	*Clostridium butyricum*	C57BL/6 male mice (18–22 g, 6–8 weeks) Three groups: 1.Control group (n: 10) 2. MPTP group (n: 10) 3.MPTP + Cb group (n: 10)	5 × 10^8^ CFU/0.2 mL/day/mice	4 weeks	Improved gut microbiota dysbiosis Colonic glucagon-like peptide-1 (GLP-1) levels vere raised Upregulated the expression of cerebral GLP-1 receptor The level of TH in SN was increased. In mice, Cb prevented dopaminergic neuronal loss caused by MPTP In the MPTP group, Cb supplementation could significantly prevent the decreased synapsin I level The effects of Cb treatment on MPTP-induced motor deficits in mice were shown to be effective	[80]
Parkinson Disease	*Lactobacillus acidophilus, Bifidobacterium bifidum, Limosilactobacillus reuteri, and Limosilactobacillus fermentum*	Male Wistar rats (weighing 200–250 g) 1.Probiotic group 2.Parkinson group 3.Sham group	Each bacteria 2 × 10^9^	14 days	MDA levels in the midbrain decreased as a result of probiotics The number of damaged neurons in the PD group was significantly lower The increase in contralateral rotations was greatly reduced by the application of a probiotic When compared to PD rats, probiotic treatment led to a decrease in escape latency Probiotics significantly prevented the memory impairment as evindenced by an increase the time spent in the target quadrant	[81]
Parkinson Disease	*Ligilactobacillus salivarius AP-32*	Male Sprague–Dawley rats (eight-weeks-old, weight 290 ± 10 g) 1.ND (non-diseased, n: 5) 2.PD (untreated PD, n: 5), 3.LD (PD treated with 8 mg of L-DOPA, n: 5) 4. 1X (PD supplemented with 1.03 × 10^9^ CFU/kg BW of probiotic, n:5), 5.MR (PD supplemented with 62 mg/kg BW of MR, n: 5), 6.1XMR (PD supplemented with a combination of 1X and MR, n:5)	0.3 × 10^9^ CFU to 0.6 × 10^9^ CFU for 300–600 g BW of rat 1.03 × 10^9^ CFU/kg BW	8 weeks	Between PD and 1X groups Increased serum SOD, glutathione peroxidase (GPx) and catalase levels, decreased ROS and TNF-α levels Increased total SCFAs, propionic and butyric leves in feces Probiotic supplementation also changed the composition of the fecal microbiota, enriching commersals while reducing some pathogenic bacreria Reduced dopaminergic neuron loss, improved endurance performance, elevated tyrosine hydroxylase (TH +) in the striatum and substantia nigra, and provided neuroprotective effects	[82]
Parkinson Disease	*Lactiplantibacillus plantarum CRL 2130,* *Streptococcus. thermophilus CRL 808,* *Streptococcus* *thermophilus CRL 807*	Eight-week-old C57BL/6 male mice (20–30 g) 1. Control 2. MIX (probiotic) 3. MPTP 4. MPTP/MIX	8 ± 2 × 10^8^ CFU/mL	22 days	When comparing the MPTP/mixture group to MPTP group, the number of tyrosine hydroxylase positive cells in the brain increased significantly MPTP-induced LAB-reduced motor deficits When compared to the MPTP group, serum TNF-α, IL-6 levels decreased and IL-10 increased significantly in the MPTP/mixture group When compared to the MPTP group, brain IL-10 increased significantly in the MPTP/mixture group	[83]
Parkinson Disease	*Lactiplantibacillus plantarum PS128 (PS128)*	Male Sprague–Dawley rats (10-week-old, ~ 400 g) 1. Saline 2. PS128 3. Levodopa 4. DBS (deep brain stimulation) 5. PS128 + Levodopa 6. PS128 + DBS 7. Levodopa + DBS	1.5 × 10^10^ CFU	6 weeks	PS128-treated rats showed a significant neuroprotective effect; there were 22,3% and 9,9% of TH + areas in the striatum and midbrain, respectively PS128 consumption inhibited the mortality of dopaminergic cell death PS128-Treated improved motor functions in hemi-parkinsonian rats PS128 administration increased brain dopamine availability in hemiparkinsonian rats	[84]
Parkinson Disease	*Bifidobacterium breve strain A1 [MCC1274] (B. breve A1)*	Male C57BL/6 mice (7–8 weeks old) 1. Control-Saline, n: 36; 2. Control-*Bifidobacterium breveA1*, n: 32; 3. Control-Non-viable *Bifidobacterium breveA1*, n: 5; 4. MPTP-Saline, n: 36; 5. MPTP- *Bifidobacterium breveA1*, n: 32; 6. MPTP-Non-viable *Bifidobacterium breveA1*, n: 5	1 × 10^9^ CFU	4 days	In Parkinson disease mice, *Bifidobacterium breve A1* restored decreased dendritic Spine Density No significant differences calcium-binding adapter molecule 1 (Iba1) and BDNF, neuropsin mRNA expression decreased Neuropsin mRNA expression decreased, while there was no significant alterations calcium-binding adapter molecule 1 (Iba1) and BDNF	[85]
Parkinson Disease	*Lacticaseibacillus rhamnosus HA-114*	Thirty-one experimentally naive adult male Sprague Dawley rats 1.Sham + Probiotics (n:12) 2.PD + Placebo (n: 9) 3. PD + Probiotics (n: 10)	10^8^ CFU	6 weeks	In 6-OHDA-Lesioned Rats, Probiotics treatment wasn’t impact anxiety behaviour There is no difference in the number of dopamine neurons in the two groups Probiotics alleviate hippocampal-dependent cognitive impairments in 6-OHDA lesioned rats	[86]

Most of the recent studies on this disease in the accumulated literature are animal studies, and studies on humans are more limited. Therefore, studies on humans are needed.

### Parkinson’s disease

Parkinson's disease is a common neurodegenerative condition marked by diminished motor abilities brought on by dopaminergic nigrostriatal system dysfunction [128]. The disorder is brought on by the selective death of dopaminergic neurons in the substantia nigra, which lowers the levels of the neurotransmitter in the striatum and may result in abnormal motor control. Bradykinesia, resting tremor, rigidity, postural instability, and muscle tone and are all motor symptoms. In addition, a few non-motor symptoms such as sleep disturbances, loss of smell, dementia, psychosis, fatigue, pain anxiety, depression, hypophonia, dysphagia, and autonomic dysfunctions may be observed in Parkinson's patients. Parkinson's patients may also experience a number of non-motor symptoms, including sleep disturbances, loss of smell, dementia, psychosis, fatigue, pain anxiety, depression, hypophonia, dysphagia, and autonomic dysfunctions [129, 130].

Parkinson's disease has a multifactorial etiology, possibly resulting from the combined effects of environmental and genetic factors. Toxic chemical exposure, head injury, environmental factors, genetic and epigenetic risk factors, and aging are the main factors associated with Parkinson's [131, 132]. Depending on these factors, factors such as α-synuclein misfolding and accumulation, oxidative stress state, decreased mitochondrial complex 1 activity, mitochondrial damage, abnormalities in adaptive and innate immune response, proinflammatory cytokines and inflammatory cell activation may affect the pathogenesis of Parkinson's disease [131]. At the same time, the renin angiotensin system is associated with Parkinson's disease. Angiotensin II is a pro-inflammatory peptide that can activate the NADPH-dependent oxidase complex, causing the formation of ROS, which may lead to the death of dopaminergic cells [133]. AT 1 and AT 2 receptors were associated with dopaminergic system [134]. Angiotensin converting enzyme activity and Angiotensin II levels were elevated by dopamin degeneration. Treatment with AT1 antagonists in rats decreased the loss of dopaminergic cells and microglial activation brought on by 6-OHDA [135]. Inhibited angiotensin II AT1 receptors led to increased D1 receptor activation, decreased in the neurotoxin-induced levels of lipid peroxidation and protein oxidation, as well as the death of dopaminergic neurons [136, 137].

Additionally to these elements, it has been revealed that the gut-brain axis may be effective in the pathogenesis of Parkinson's Disease. It has been shown that there is a change in the microbiota of Parkinson's patients. In a meta-analysis study, it was observed that *Akkermansiaceae* and *Catabacter* levels increased, *Roseburia*, *Faecalibacterium* and *Lachnospiraceae* ND3007 levels decreased in Parkinson's patients [138]. Studies have shown changes in many bacterial strains in the case of Parkinson's disease, and their levels have increased or decreased (Table 4). These individuals also experienced alterations in their microbiota, as well as carbohydrate fermentation, a reduction in butyrate synthesis ability, proteolytic fermentation, and the development of dangerous amino acid metabolites such p-cresol and phenylacetylglutamine [139–145]. Changes in the microbiota have also been associated with symptoms. Bacteroides levels were increased more in Parkinson patients without tremor than in patients with tremor. Microbiota change has been associated with increased levels of IFN-γ and TNF-α. Increase in *Bacteroides* level was associated with TNF-α, increase in *Verrucomicrobia* strain was associated with IFN-γ [142]. It has been found that fecal branched-chain amino acid levels and aromatic amino acid concentrations are decreased in individuals with Parkinson's disease [145]. Considering the changes in the microbiota, studies have shown that SCFAs (acetate, butyrate, propionate) levels decrease in Parkinson's patients. In particular, it has been demonstrated that there is a decrease in propionate levels [146–148].

**Table 4:** Potential change in microbiota composition in Parkinson's condition

Increments	Descendants	Reference
*Verrucomicrobiaceae, Bifidobacteriaceae, Bifidobacterium, Streptococcaceae, Desulfohalobiaceae, Akkermansia, Escherichia, Prosthecobacter, Streptococcus, Clostridium, Serratia, Enterobacter*	*Bacteroidaceae, Lachnospiraceae, Brevibacteriaceae, Sphingobacteriaceae, Bacteroides, Brevibacterium,Blautia, Odoribacter, Lachnospira, Butyrivibrio, Roseburia, Pseudobutyrivibrio, Dolichospermum, Coprococcus,*	[139]
*Christensenellaceae, Desulfovibrionaceae, Bifidobacterium, Bilophila, Akkermansia*	*Lachnospiraceae, Faecalibacterium*	[140]
*Christensenella, Catabacter, Lactobacillus, Oscillospira, Bifidobacterium, Christensenella minuta, Catabacter hongkongensis, Lactobacillus mucosae, Ruminococcus bromii, and Papillibacter cinnamivorans*	*Dorea, Bacteroides, Prevotella, Faecalibacterium, Bacteroides massiliensis, Stoquefichus massiliensis, Bacteroides coprocola, Dorea longicatena, Coprococcus eutactus, Ruminococcus callidus, Blautia glucerasea, Prevotella copri, Bacteroides dorei, Bacteroides plebeus*	[141]
*Verrucomicrobia, Mucispirillum, Porphyromonas, Lactobacillus, Parabacteroides*	*Prevotella*	[142]
*Clostridium XVIII, Clostridium IV, Sphingomonas, Butyricicoccus Holdemania, Aquabacterium, Anaerotruncus*	*Lactobacillus, Sediminibacterium*	[143]
*Lactobacillaceae Lactobacillus* *Lachnospiraceae* *NK4A136, Bifidobacteriaceae Bifidobacterium, Desulfovibrionaceae* *Bilophila, Lachnospiraceae Tyzzerella*	*Lachnospiraceae Blautia* *Lachnospiraceae Fusica-* *tenibacter*	[144]
*Rikenellaceae_RC9_gut_group, Bifidobacterium, Parabacteroides, Actinobacteria,* *Bacteroidetes*	*Faecalibacterium*	[145]

Metabolites, neurotransmitters, vitamins, hormones, pathogenic peptides, cytokines, and neurotoxins secreted as a result of microbiota can be associated with Parkinson's Disease. Intestinal bacteria can synthesize various neurotransmitters such as GABA, 5-hydroxytryptamine (5-HT), dopamine or SCFAs [149]. Increased intestinal permeability and dysbiosis in the microbiota cause an increase in systemic inflammation (CRP, IL-6, TNF-α, IL-1β), changes in SCFAs, decrease in neurotransmitters such as GABA, and change in T-reg cell expression [150]. Through the gut-brain axis, intestinal dysbiosis can result in an increase in T helper cells, proinflammatory cytokines, and LPS, resulting in increased intestine and blood–brain barrier permeability [151]. In case of disruption of the crostalk in the intestinal-brain axis, abnormal α-synuclein fibrils can accumulate in the ENS, glial cell dysfunction, and inflammation in the ENS can occur. Increased penetration of microbiota-generated substances across the blood–brain barrier is associated with an increase in Parkinson's disease symptoms through the death of dopaminergic neurons, neuroinflammation, and an increase in α-synuclein [152].

Synucleinopathy involving the accumulation of insoluble polymers of α-synuclein with Lewy bodies proteins has been found in Parkinson's patients. Lewy bodies reduce neuronal growth and cause neurodegeneration [149]. Alterations in the gut microbiota result in aberrant products that have toxic effects on the peripheral gut ganglia and lead to an excessive synthesis of α-synuclein. α-synuclein uses the medulla oblongata, vagus nerve, and brain stem to reach the cortex, thereby affecting damage to neurons in the central system [153].

A decrease in SCFAs may occur in patients with Parkinson's disease [146–148]. SCFAs are effective in preventing dopaminergic neuronal loss, reducing neuroinflammation, regulating microglia function, preserving blood–brain barrier function, regulating the growth, differentiation and survival of synapses, regulating neurotropic factor secretion (BDNF, GDNF glial-derived neurotropic factor), maintaining the intestinal barrier by down-regulating TLR expression, decreasing proinflammatory cytokines, increasing anti-inflammatory cytokine production and decreasing oxidative stress [154]. It has been found that propionate supplementation regulates zonula occludens-1 and occludin and has a positive effect on motor behavior and intestinal epithelial barrier through Akt signaling pathway [147]. In addition, it was stated that as a result of FMT, microbiota dysbiosis decreased, fecal SCFAs increased, physical disorders were alleviated, and dopamine and serotonin levels increased in Parkinson's patients [155].

For all these reasons, it has been emphasized that probiotic supplementation may have positive effects in Parkinson's patients. It has been stated that increasing the protection of dopaminergic neurons, reducing pain, inflammation, behavioral changes and oxidative stress can be achieved by reducing gastrointestinal motility, pathogenic bacteria and gastrointestinal abnormalities [156].

**Fig. 3:**
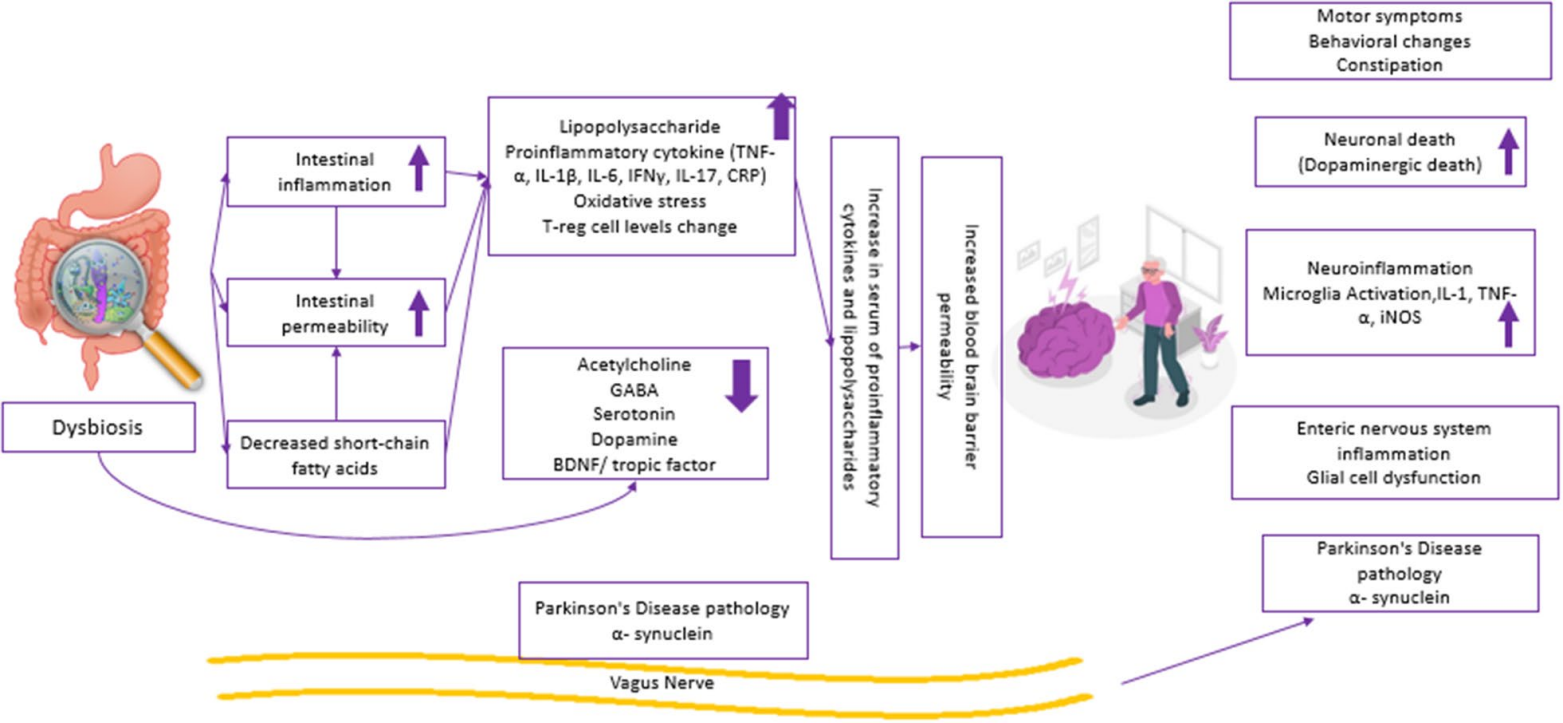
Possible effects of dysbiosis in the microbiota in Parkinson's disease

In Fig. 3, the relationship between dysbiosis in the microbiota and Parkinson's disease is summarized.

**Fig. 4:**
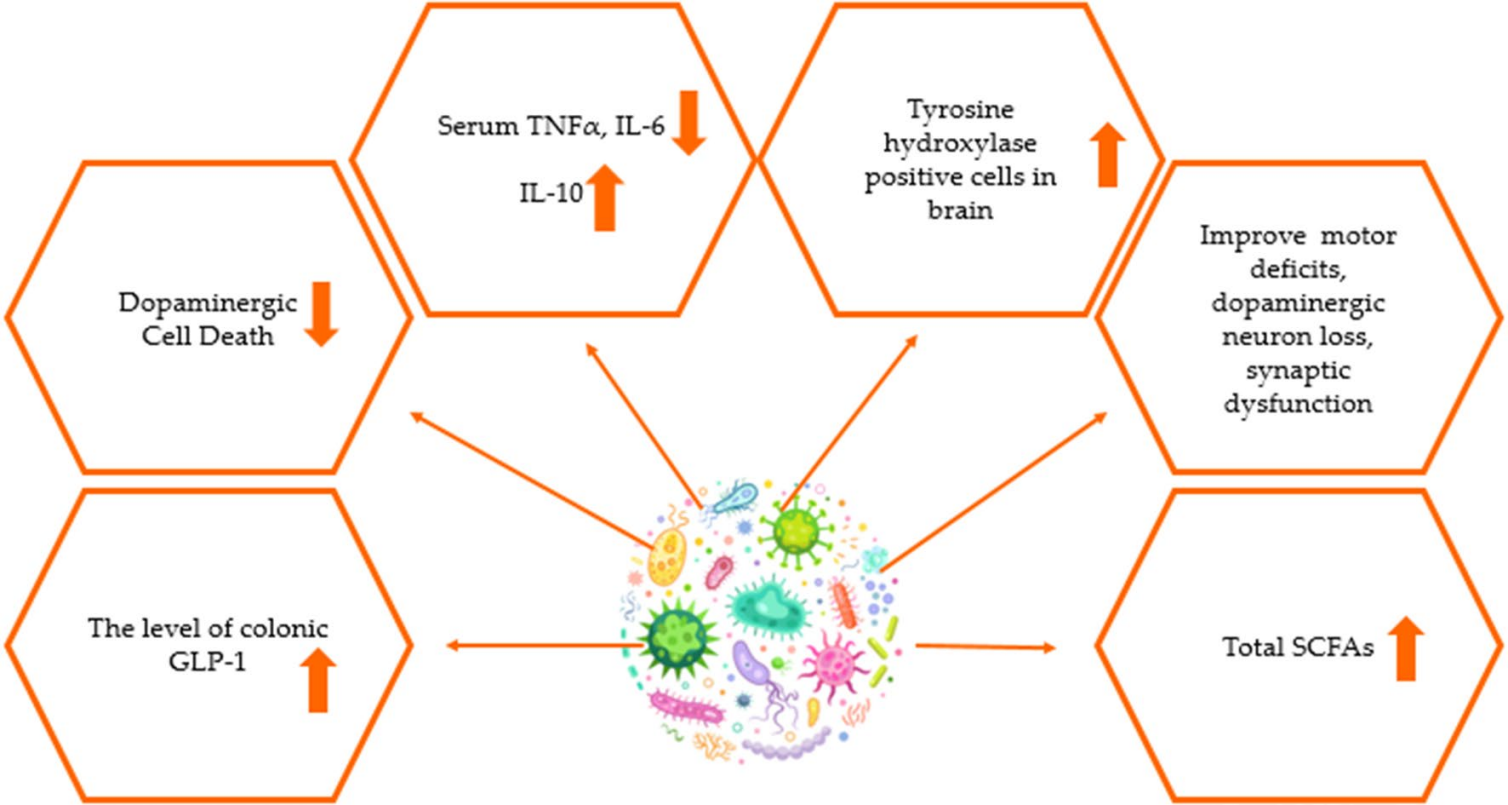
Some potential effects of LBPs supplementation in Parkinson's disease

Human and animal studies examining the effect of probiotic supplementation for Parkinson's disease are listed in Table 2 and Table 3. Generally, *Lactobacillus* and *Bifidobacterium* strain bacteria were used in both study types. Probiotic supplementation showed effects on motor activity, BDNF level, microbiota dysbiosis, synaptic dysfunction, memory, inflammation, oxidative stress, cognitive performance, dopaminergic cell death, SCFAs levels. Figure 4 summarizes the general effects of probiotic supplementation in Parkinson's patients [69–71, 80–86]. Although there are animal studies to elucidate the mechanisms in Parkinson's disease, human studies on probiotic supplementation in recent years are limited. In order to assess the impact of supplementation in humans, it is crucial to expand the number of research.

## Future conflicts and safety

**Fig. 5:**
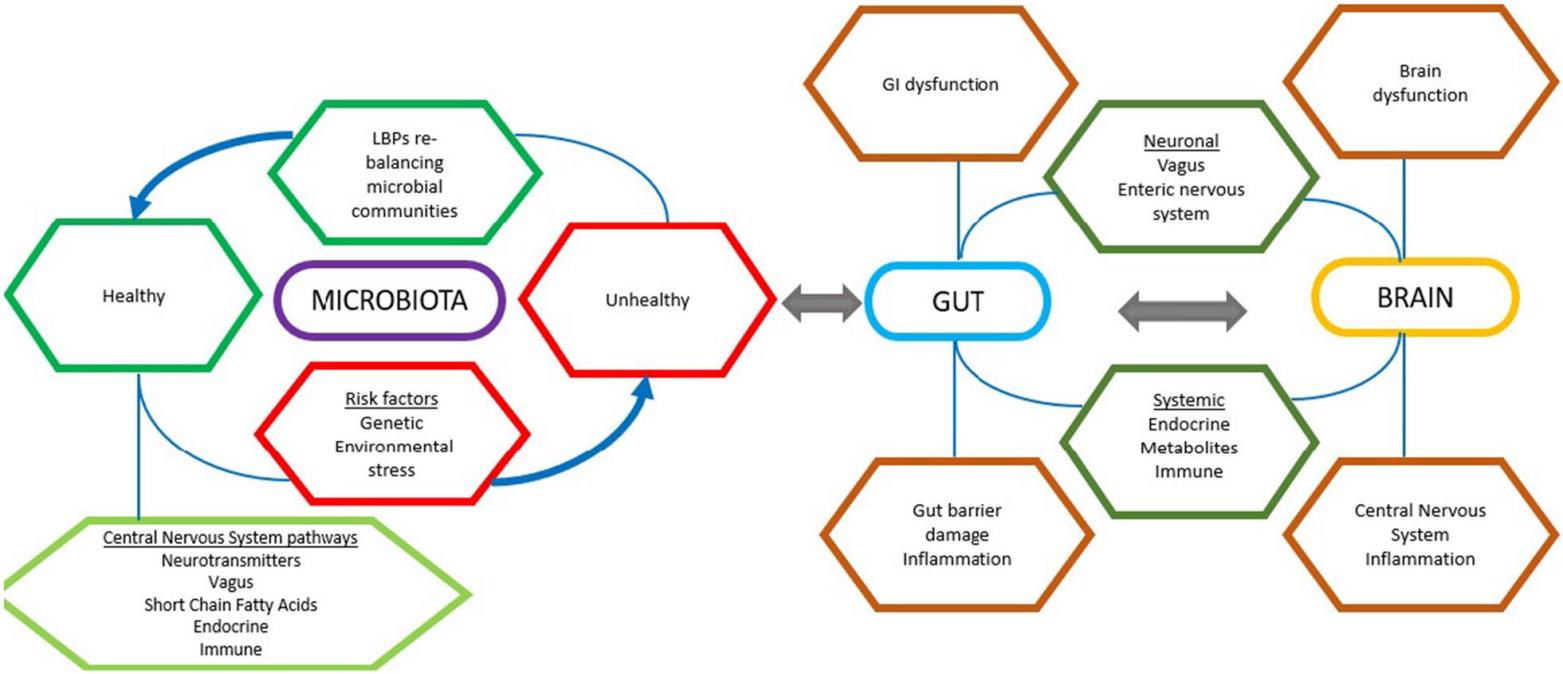
LBPs and gut-brain interaction (adapted from reference [26])

LBPs often do not exert their biological effects by reaching distant organs, tissues, or receptors or by acting directly on a defined target. Instead, they have an impact on the host microbiota through suppressing pathogens [157], generating active molecules/metabolites [65, 74, 158, 159], by modulating mucosal immune system activity [160, 161] or by modulating nervous system activity [77, 79, 158, 162]. These effects could all or part of them happen simultaneously, mediating various sorts of signals and activating distinct physiological pathways within the host. There are several interconnected systems in which LBPs can potentially alter brain function directly and/or indirectly (Fig. 5). These include specifically the endocrine [163], parasympathetic autonomic (vagus nerve) [164] and immune system [165]. All these situations can also affect cognitive and behavioral processes, which can cause behavioral changes [41].

One of the most important points is to demonstrate the quality, efficacy and safety of LBPs due to the product's vivid properties and often multifactorial mode of action. Thus, the global profit-risk ratio can be evaluated by determining the features and risks of the product components and strains, as well as the characteristics and risks of the target population [12, 166].

The risks to be considered in the LBP risk analysis regarding the patient are the risks associated with the ingestion of the live product. These include nature of the target, mode of action, dosage, formulation, route of administration, biodistribution in the intended population, pathophysiology, patient's characteristics (age, gender, genetics, ethnicity, microbiome composition, environment, lifestyle, diet), special populations associated risks (pregnant, premature babies, children, elderly, critically illness), patient’s concomitant medication, risk for entourage. Non-clinical toxic/safety studies that take into account the risks associated with the targeted population (in vitro, ex vivo, methods/models developed with appropriate animal models) and then first in human/early clinical trials are required. If one or more of the risks revealed by the risk analysis is affected by dosage, toxicity studies should include multiple dosages to provide and document information for human translation. Contingency plan should be developed the event of serious negative effects on the target population [166].

## Conclusions

Recent advances in human and animal studies revealed that the gut microbiome and especially dysbiosis can cause mood disorders, neurodevelopmental and neurodegenerative conditions by communicating the gut-brain axis to communicate with the brain. LBPs, a recent emerging class of therapeutics based on probiotics and live bacterias, are promising for preventing and treating these gut brain axis related conditions and heath problems. Although there is increasing evidence to suggest that LBPs have a stabilizing effect on the gut-brain axis [65, 74, 77, 79, 157–162] results are not consistent because of lack of control for certain variables used in studies, such as strain, dose, length of treatment, placebo control, sample size, mixed male/female patient population, and other study design issues, are major barriers in this regard.

Disease-specific probiotic strains need to be identified. The duration of probiotic dose administration and the monitoring of the results of probiotic use should also be taken into account at the same time. The quality of the studies will be improved by integrating information on food consumption of people whose effects on the gut microbiota are known (for self-human studies), in addition to the probiotics employed in the experiments. Gender should also be emphasized for each condition in order to determine whether it affects the disease or not. Future research can remark on the usage of probiotics specific to the disease and gender in this direction. Additionally, it's believed that standardizing the scales used in research to assess disease and symptom indicators will be helpful in assessing the impact of probiotics on the disease. Finally, comparing the study's findings to those from the biological samples will aid in understanding how probiotics affect disease and metabolism.

Regarding safety, the general opinion is that commonly used strains such as *Lactobacillus* and *Bifidobacteria* are safe. More thorough safety and efficacy studies will be required as the field develops and a greater range of possibilities when novel therapies begin to be examined. There is a need for more multiple and specific target studies to be carried out considering all risks to elucidate the mechanism and strain specificity. The publication of subject-specific guidelines and public–private collaboration and extensive partnerships are required first to identify and develop LBPs that can be successful as specific therapeutics for safe and specific modulation of the gut microbiota-brain axis.

## Data Availability

Not applicable.
